# Digenic Inheritance: Evidence and Gaps in Hemophagocytic Lymphohistiocytosis

**DOI:** 10.3389/fimmu.2021.777851

**Published:** 2021-11-17

**Authors:** Erica A. Steen, Michelle L. Hermiston, Kim E. Nichols, Lauren K. Meyer

**Affiliations:** ^1^ University of California, San Diego, San Diego, CA, United States; ^2^ Department of Pediatrics, University of California, San Francisco, San Francisco, CA, United States; ^3^ Department of Oncology, St. Jude Children’s Research Hospital, Memphis, TN, United States

**Keywords:** hemophagocytic lymphohistiocytosis, digenic, degranulation, variants, cytotoxic lymphocyte, natural killer cell

## Abstract

Hemophagocytic lymphohistiocytosis (HLH) is a hyperinflammatory disorder characterized by the inability to properly terminate an immune response. Familial HLH (FHLH) and related immune dysregulation syndromes are associated with mutations in the genes *PRF1, UNC13D, STX11, STXBP2, LYST, AP3B1*, and *RAB27A*, all of which are required for the assembly, exocytosis, and function of cytotoxic granules within CD8+ T cells and natural killer (NK) cells. Loss-of-function mutations in these genes render the cytotoxicity pathway ineffective, thereby failing to eradicate immune stimuli, such as infectious pathogens or malignant cells. The resulting persistent immune system stimulation drives hypercytokinemia, ultimately leading to severe tissue inflammation and end-organ damage. Traditionally, a diagnosis of FHLH requires the identification of biallelic loss-of-function mutations in one of these degranulation pathway genes. However, this narrow definition fails to encompass patients with other genetic mechanisms underlying degranulation pathway dysfunction. In particular, mounting clinical evidence supports a potential digenic mode of inheritance of FHLH in which single loss-of-function mutations in two different degranulation pathway genes cooperate to impair pathway activity. Here, we review the functions of the FHLH-associated genes within the degranulation pathway and summarize clinical evidence supporting a model in which cumulative defects along this mechanistic pathway may underlie HLH.

## Introduction

Hemophagocytic lymphohistiocytosis (HLH) is a hyperinflammatory syndrome mediated by an ineffective yet hyperactive immune response. Patients with HLH appropriately initiate an immune response in the presence of an immunogenic stimulus, but they display impaired eradication of these stimuli, resulting in persistent immune cell activation and excessive cytokine secretion. This in turn can lead to fatal end-organ damage in the absence of treatment aimed at controlling hyperinflammation (1). Based on diagnostic criteria proposed by the Histiocyte Society in conjunction with the HLH-2004 clinical trial, HLH should be considered in patients presenting with at least five of eight clinical features including fever, splenomegaly, multi-lineage cytopenias, hypofibrinogenemia or hypertriglyceridemia, hyperferritinemia, hemophagocytosis, elevated soluble CD25, and low or absent natural killer (NK) cell activity (2).

Historically, HLH has been subdivided into primary, or familial HLH (FHLH), and secondary, nonfamilial HLH (3, 4). FHLH most commonly presents during infancy or early childhood, with approximately 70% of patients presenting before one year of age (3). Inherited in an autosomal recessive manner, FHLH is characterized by the presence of germline homozygous or compound heterozygous loss-of-function (LOF) mutations in a defined set of FHLH-related genes consisting of *PRF1, UNC13D, STX11*, and *STXBP2*, which comprise FHLH subtypes 2-5, respectively. Related immunologic disorders characterized by germline biallelic LOF mutations in genes such as *LYST, AP3B1*, and *RAB27A* similarly underlie the development of HLH. Each of these genes is essential to the cytolytic activity of cytotoxic T-lymphocytes (CD8+ T-cells, hereafter referred to as CTLs) and NK cells (2). In contrast, secondary HLH has historically been diagnosed in patients without a clear genetic predisposition to immune dysregulation. Most commonly presenting in later childhood or adulthood, these patients develop hyperinflammation following exposure to a strong immunogenic stimulus such as an infection or malignancy (4). Many reports have demonstrated that homozygous LOF variants affecting FHLH genes result in early disease presentation, sometimes without an identifiable trigger (5). However, patients harboring less damaging variants, such as missense alterations that impair but do not eliminate protein expression or function, may also develop disease at a later age or in response to a more significant immunogenic stimulus (5). Thus, familial and secondary HLH are often challenging to differentiate in the absence of germline genetic testing.

In addition to the classic monogenic model of autosomal recessive inheritance as a cause for FHLH, there is increasing evidence in favor of a mechanism mediated by digenic inheritance (DI), defined broadly as germline genetic variation at two distinct loci that cooperate to mediate disease (6). Practically, this definition is more nuanced, as there is a spectrum of the extent to which two co-inherited variants interact. As a result, it has been proposed that DI be subclassified into pseudo-DI and true DI. In pseudo-DI, the inheritance of one pathogenic variant is itself sufficient to cause disease. However, the phenotype may be modified by co-inheritance of a second pathogenic variant that when present, enhances disease severity. In this scenario, the disease may be considered monogenic but with a variable phenotype determined by the presence of another variant that functions as a genetic modifier. True DI, in contrast, occurs when mutations in two different genes are required for disease to occur, representing oligogenic inheritance (7). In this review, we discuss the functions of genes associated with FHLH and related immunologic disorders and describe the consequences resulting from LOF mutations in those genes, providing a framework for considering how LOF mutations in two distinct genes within the same functional pathway may cooperate to mediate disease. We then review the clinical data suggesting potential DI and consider areas in which additional research is necessary to better understand the functional implications of this genetic mechanism.

## Biology of the Degranulation Pathway

**Figure 1:**
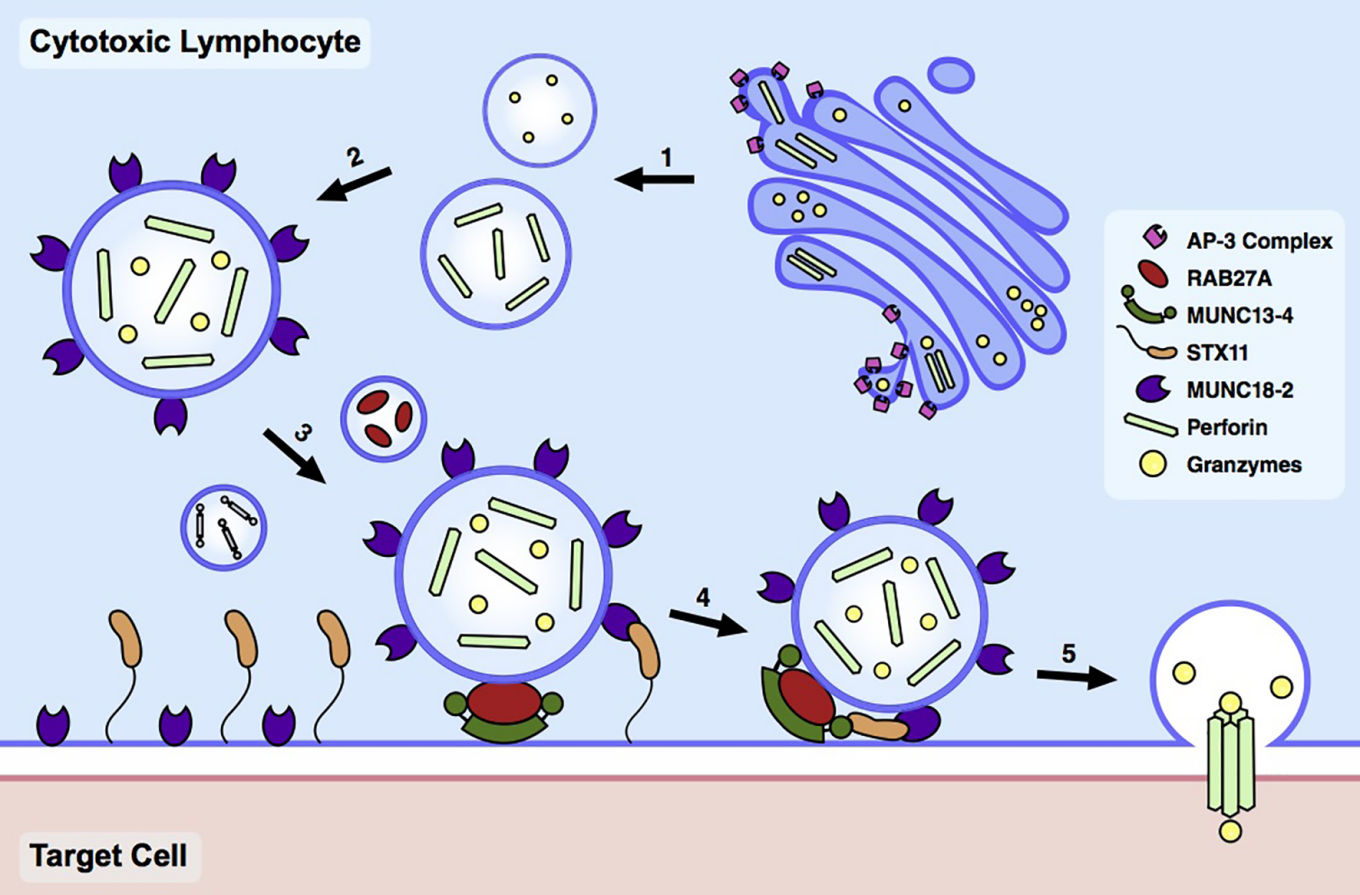
Cytotoxic lymphocyte degranulation pathway. Upon target cell engagement, the degranulation pathway in cytotoxic lymphocytes undergoes a series of coordinated steps consisting of (1-2) cytotoxic granule biogenesis, (3-4) docking and priming, and (5) fusion and pore formation.

CTLs and NK cells respond to and eliminate infected or malignant cells *via* the release of cytotoxic granules (CGs) at the immunological synapse (IS) formed at the site of target cell engagement (8). These CGs contain serine proteases, known as granzymes, which enter the target cell and induce caspase-dependent and -independent apoptosis (9). Successful target cell killing *via* the degranulation pathway requires the coordinated activity of numerous proteins involved in granule biogenesis, trafficking, and exocytosis (**Figure 1**). Homozygous or compound heterozygous LOF mutations in any one of these respective genes underlie HLH and related immune dysregulation syndromes by impeding the ability to terminate the immune response (**Table 1**). Here, we briefly review the molecular and biochemical functions of these genes, focusing on the consequences of LOF mutations. For more detailed information about protein function, we refer the reader to several excellent reviews on this topic (9–12).

**Table 1:** Degranulation Pathway Genes in HLH and Related Immune Dysregulation Syndromes.

Disease	Gene	Protein	Primary Function
Chediak-Higashi Syndrome	*LYST*	LYST	Biogenesis
Hermansky-Pudlak Syndrome	*AP3B1*	β3A subunit of AP-3 complex	
Griscelli Syndrome Type 2	*RAB27A*	RAB27A	Docking
Familial HLH Type 3	*UNC13D*	MUNC13-4	Priming
Familial HLH Type 4	*STX11*	STX11	Fusion
Familial HLH Type 5	*STXBP2*	MUNC18-2	
Familial HLH Type 2	*PRF1*	Perforin	Delivery

Summary of the degranulation pathway genes and associated proteins underlying FHLH and related immune dysregulation syndromes.

### Biogenesis

CGs are specialized secretory lysosomes that are assembled in the cytoplasm of CTLs and NK cells. Granzymes and other granule contents are trafficked from the Golgi network to the core of these lysosomes, where an acidic microenvironment maintains the cytotoxic proteins in an inactive state prior to their release at the IS. Two key effectors of proper CG biogenesis are the lysosomal trafficking regulator (LYST) and the clathrin adaptor protein 3 (AP-3) complex (9).

Biallelic LOF mutations in *LYST* underlie Chediak-Higashi syndrome (CHS), a rare inherited immune disorder in which patients can develop the signs and symptoms of HLH. The clinical features of CHS, including oculocutaneous albinism, prolonged bleeding, neurodegeneration, and immune dysregulation, are all attributable to widespread secretory granule dysfunction in multiple cell types. In particular, the impact of these mutations on immune cell function predisposes to hyperinflammation, which is the primary cause of mortality for patients with CHS (13). Functionally, CTLs and NK cells from patients with CHS are characterized by the presence of fewer but significantly enlarged CGs relative to wild-type (WT) cells (9). In WT CTLs, CGs are undetectable prior to exposure to an immune stimulus. Upon CTL activation, cytotoxic protein expression is rapidly upregulated, concomitant with the assembly of numerous, small CGs in the cytoplasm (14). In contrast, in CHS CTLs, CGs initially form identically to those in WT CTLs, but at the later stages of CTL activation, can be seen forming giant intracellular structures (14).

*LYST-*deficient CTLs and NK cells display impaired target cell killing, suggesting that these giant CGs are unable to successfully release cytotoxic proteins at the IS. Gil-Krzewska et al. studied NK cells from patients with CHS carrying mutations in the ARM/HEAT (armadillo/huntingin/elongation factor 3, protein phosphatase 2A, TOR1) domain of *LYST*, a functional domain involved in vesicular trafficking. These cells had fewer and larger CGs relative to WT NK cells. While these large CGs appropriately localized to the IS following target cell engagement, they were unable to fuse with the plasma membrane, thereby preventing the release of cytotoxic proteins toward the target cell (15). To further study the functional consequences of these ARM/HEAT domain mutations, the same authors used CRISPR-Cas9 genome editing to mutate *LYST* in a human NK cell line. As observed in the patient cells, these knockout cells had a small number of large CGs. In order for CGs to fuse with the plasma membrane to release their contents, they must traverse a network of cortical actin that accumulates at the IS. These authors demonstrated that the formation of this network was identical in WT and knockout cells, however the enlarged granules from the *LYST-*deficient cells were excluded from the small openings within the actin meshwork, impeding their localization to the plasma membrane. Treatment of these cells with compounds that interfere with actin polymerization potentiated degranulation and restored cytotoxicity, indicating that CGs in *LYST-*deficient cells retain functionality but are prevented from reaching the cell surface as a result of their excessively large size (16).

The AP-3 protein complex is involved in the formation of clathrin-coated vesicles that traffic to and from lysosome-related organelles (17), a process that is essential for CG biogenesis. Hermansky-Pudlak syndrome (HPS) is a collection of ten autosomal recessive immune disorders mediated by LOF mutations in the subunits of this protein complex. HPS shares a number of clinical features with CHS, including albinism, a bleeding diathesis, and immune dysregulation, reflecting a shared underlying pathophysiology related to secretory lysosome dysfunction. In particular, the immune dysregulation in patients with HPS type 2 (HPS2), caused by biallelic LOF mutations in the β3A subunit (*AP3B1*) of the AP-3 complex, has been associated with the development of HLH (18).

Cells from patients with HPS2 have decreased protein expression of all AP-3 complex subunits relative to WT cells, as the β3A subunit is thought to stabilize this multi-protein complex. LOF mutations in this subunit alter the conformation of the complex and increase its susceptibility to proteolytic degradation (19). As a result, cells from patients with HPS2 lack the function of the entire AP-3 complex. While LYST regulates the size of CGs, the AP-3 complex is necessary for the appropriate localization of key lysosomal proteins to these granules, which are in turn required for their function. Specifically, this complex mediates the shuttling of proteins from the Golgi network to the lysosome (9). Studies using fibroblasts (20), CTLs (21), and NK cells (22) have consistently demonstrated increased mis-localization of lysosomal proteins to the plasma membrane in cells from patients with HPS2.

### Docking and Priming

Following their transport along microtubule pathways to the IS, CGs must dock at the plasma membrane and undergo a priming step prior to their fusion with the membrane and subsequent exocytosis of their contents. Critical mediators of these processes include RAB27A and MUNC13-4, respectively (9).

Griscelli syndrome type 2 (GS2) is an autosomal recessive immune disorder characterized by biallelic LOF mutations in *RAB27A*, a gene encoding a small GTPase. As in CHS and HPS2, patients with GS2 present with albinism, neurologic sequelae, and immune dysregulation frequently associated with the development of HLH (23). In regards to the latter, CTLs from patients with GS2 produce CGs with appropriate contents, but these granules are unable to be released *via* exocytosis, correlating with impaired cytotoxicity (24).

Using CTLs from *ashen* mice, a murine model of human GS2 in which animals lack *Rab27a* expression, Stinchcombe et al. studied the mechanistic basis for the cytotoxicity defect in this disease. The authors demonstrated that upon target cell engagement, CGs in RAB27A-deficient CTLs appropriately migrated along microtubules to re-localize to the IS. However, using electron microscopy to obtain detailed images of the IS, they found that these properly localized granules failed to dock to the membrane, with a subsequent failure to release their contents into the IS (25).

Similar to the phenotype in cells from patients with GS2, CTLs and NK cells from patients with FHLH3 demonstrate impaired cytotoxicity despite appropriate localization of CGs to the IS following cell activation. FHLH3 is characterized by biallelic LOF mutations in *UNC13D*, which encodes the MUNC13-4 protein. Like RAB27A, the vesicular distribution of MUNC13-4 in resting CTLs is largely distinct from that of granzymes. Following cell activation and formation of the IS, vesicles containing RAB27A, vesicles containing MUNC13-4, and CGs co-localize at the IS and fuse into a common vesicular structure (26). While RAB27A mediates the docking of these CGs, MUNC13-4, itself tethered to the membrane *via* a required protein-protein interaction with the small GTPase RhoG (27), is required for the final step prior to granule fusion with the plasma membrane. This final process, known as priming, makes these granules competent for exocytosis (9). As a result, CGs in MUNC13-4-deficient CTLs can be seen docking at the plasma membrane, but failing to undergo exocytic fusion (28).

Much of what is known about the priming function of MUNC13-4 at the IS comes from studies of the role of other MUNC13 family members in regulating neurotransmitter release at neurological synapses. Using total internal reflection fluorescence microscopy (TIRFM) in neuroendocrine cells, it has been shown that following MUNC13-mediated priming, vesicles docked at the plasma membrane have significantly reduced mobility (29). Similarly, in activated MUNC13-4-deficient murine CTLs, the docked CGs are significantly more mobile than those in WT CTLs, and this phenotype can be rescued with ectopic expression of MUNC13-4 (30).

### Fusion

After docking and priming, CGs are finally able to fuse with the plasma membrane, enabling the release of their cytotoxic contents across the IS toward the target cell. This fusion process is mediated by the activity of soluble N-ethylmaleimide-sensitive factor attachment protein receptors (SNAREs), a family of proteins that are ubiquitous through the immune system, where they function to mediate the fusion of docked vesicles with their target membranes. While the requirement for specific SNARE proteins differs according to cell type, the mechanism is shared for all vesicular fusion events. Specifically, SNARE proteins on the vesicular membrane lock together with SNAREs on the target cell membrane to form a protein bridge between the two structures. This interaction then pulls the two compartments into close proximity, and a subsequent conformational change in the SNARE protein complex generates a force that is sufficient to fuse the lipid bilayers of the two membrane compartments (31). These SNARE protein interactions are further regulated by the activity of SNARE accessory proteins, as binding to these accessory proteins is required for SNARE-mediated vesicle fusion with the plasma membrane (9).

LOF mutations in the genes *STX11*, encoding the SNARE protein syntaxin 11, and *STXBP2*, encoding the SNARE accessory protein MUNC18-2, underlie FHLH4 (32) and FHLH5 (33), respectively. Like patients with FHLH3, patients harboring biallelic *STX11* and *STXBP2* mutations demonstrate impaired CTL and NK cell degranulation with an associated cytotoxicity defect, despite appropriate mobilization of CGs to the IS following cell activation (33, 34). Analyzing lymphocytes from patients with FHLH4 and FHLH5, Côte et al. highlighted the importance of interactions between these two proteins. Specifically, in cells lacking MUNC18-2, they found markedly reduced expression of STX11, suggesting that MUNC18-2 is required for STX11 stabilization. The converse was not true, as MUNC18-2 expression was the same in WT and FHLH4 lymphocytes (33). This finding is further supported by work from Dieckmann et al. demonstrating a role for MUNC18-2 as a chaperone of STX11 at the plasma membrane. Using healthy human CTLs, they demonstrated that MUNC18-2 localizes primarily to CGs, traveling with them to the IS upon target cell engagement. In contrast, STX11 was found primarily at the plasma membrane, and became concentrated at the IS after cell activation. While this localization of MUNC18-2 was unchanged in the absence of STX11, STX11 was lost from the plasma membrane in cells lacking MUNC18-2 expression (35), suggesting that MUNC18-2 is required both for the stability and the proper subcellular localization of STX11. Intriguingly, in addition to its chaperone capacity, MUNC18-2 may also play a functional role in the fusion of CGs with the plasma membrane. Spessott et al. demonstrated that STX11, while anchored to the plasma membrane, can support the exchange of lipids between vesicular compartments, but cannot independently facilitate the exchange of vesicular contents. With the addition of WT MUNC18-2, but not a mutant incapable of binding to STX11, complete fusion could be induced (36). These data indicate the importance of an intact molecular interaction between STX11 and MUNC18-2 to facilitate CG fusion, thereby underscoring the defective cytotoxicity in cells from patients with either FHLH4 or FHLH5.

### Delivery

Once CGs are released from the CTL or NK cell, they traverse the IS to induce target cell apoptosis. While the granzyme contents of the CGs directly mediate apoptosis, their activity is dependent on perforin (*PRF1*), a pore-forming protein that is required for the delivery of granzymes into the target cell cytoplasm (37). The importance of perforin for the cytotoxic activity of CTLs and NK cells is reflected in the severity of the clinical phenotype associated with LOF mutations in *PRF1*, which was the first identified FHLH gene. Patients with biallelic *PRF1* mutations, now known as FHLH2, commonly present early in life with severe immune dysregulation (38).

Given the clinical significance of impaired PRF1 function, there has been considerable interest in understanding how PRF1 mediates granzyme access to the target cell cytoplasm. It is well-established that the pore-forming capabilities of PRF1 are dependent on its oligomerization into a complex that has structural similarity to the membrane attack complex formed in the innate immune system (39). Using human cell lines, Keefe et al. demonstrated that exposing cells to PRF1 induces rapid and profound changes to the plasma membrane, with the formation of membrane blebs and a loss of membrane integrity that facilitates the translocation of cell impermeable dyes into the cytoplasm. Intriguingly, when they tracked the localization of these dyes, they observed that, rather than disseminating into the cytosol, they remained contained within membrane-proximal blebs. Similarly, when cells were incubated in the presence of granzyme B and exposed to PRF1, granzyme B could be found within vesicular structures in the target cell cytoplasm, suggesting that PRF1 mediates entry into target cells *via* an endocytic process (40). These same authors went on to demonstrate that these endocytic structures, termed “gigantosomes”, contain both PRF1 and granzymes. Approximately 15 minutes after their formation, these structures rupture due to PRF1-induced pore formation in the endosome membrane. This then releases endosomal contents, thereby exposing the target cell cytoplasm to apoptosis-inducing granzymes (41).

## A Threshold Model of Genetic Predisposition to HLH

**Figure 2:**
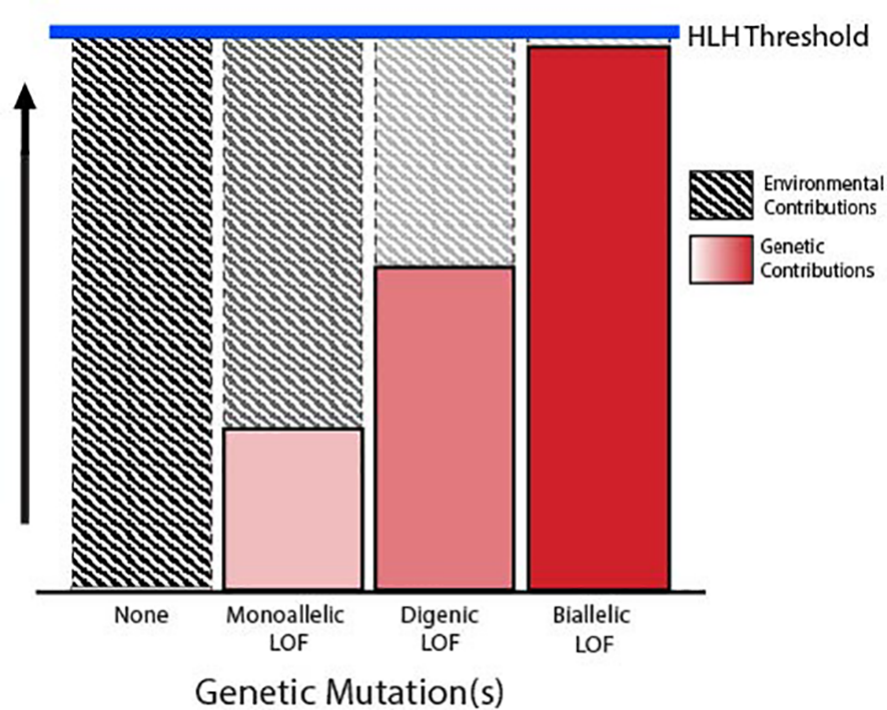
Threshold model of HLH. Schematic depicting the inverse relationship between the degree of degranulation pathway dysfunction conferred by germline variants and the environmental trigger required to reach the threshold for development of HLH.

FHLH and related immune disorders associated with an HLH phenotype have traditionally been defined as autosomal recessive diseases characterized by germline biallelic LOF mutations in one of the degranulation pathway genes, which in turn confer defective lymphocyte cytotoxicity. This is in contrast to secondary HLH, which is diagnosed in patients meeting the clinical diagnostic criteria for HLH in the absence of a clear genetic predisposition. However, there is increasing evidence that this dichotomous definition is likely an oversimplification, as it does not adequately address the complex interactions between genetic predisposition and environmental influence. A more comprehensive model considers a threshold for disease development, in which severe inherited dysfunction in the degranulation pathway is sufficient to mediate disease with little or no identifiable environmental trigger, while more subtle insults to pathway function require a profound immunogenic stimulus to induce hyperinflammation (42–44). This model suggests that a multitude of genetic mechanisms may contribute to the risk of developing HLH by mediating some degree of underlying dysfunction in the lymphocyte degranulation pathway (**Figure 2**).

Even amongst those patients with a classic autosomal recessive pattern of inheritance, clinical and experimental data suggest that LOF mutations in different degranulation pathway genes have non-equivalent consequences, with loss of some proteins resulting in a more severe phenotype than others. In a cohort of patients with complete LOF of *PRF1, RAB27A*, or *STX11*, it was found that the severity of disease differed as a function of the involved gene. At one extreme, patients with *PRF1* mutations presented with severe, early-onset disease, while those with *STX11* mutations had milder disease and presented at a later age (45). These clinical data are recapitulated in murine models of degranulation pathway dysfunction. When Jessen et al. infected multiple HLH-prone murine models with lymphocytic choriomeningitis virus (LCMV) to stimulate the development of HLH, they observed a spectrum of disease severity that correlated with the extent of the cytotoxicity defect conferred by the respective gene knockout. Based on this analysis, they proposed a hierarchy of degranulation pathway genes, with mutations in *Ap3b1* conferring the mildest phenotype followed by *Lyst, Stx11, Rab27a*, and finally *Prf1.* Interestingly, when they analyzed a cohort of patients with complete LOF of these same genes, they found the same hierarchy reflected in the average age of disease onset (46), suggesting that even amongst patients with biallelic LOF mutations, a threshold model of disease may apply.

Beyond this autosomal recessive mechanism, clinical evidence also suggests that inheritance of a single heterozygous mutation in a degranulation pathway gene may be sufficient to predispose to HLH. Zhang et al. reported on two unrelated teenagers with HLH who were found to have the same heterozygous missense mutation in *RAB27A.* When this mutation was expressed in an NK cell line, the authors found impaired degranulation and cytotoxic activity as well as decreased capacity for binding to MUNC13-4. Interestingly, clinical NK cell studies in one of these patients similarly revealed impaired NK cell function. Her father was found to be the carrier of the mutation and had a comparable degree of NK cell dysfunction but was otherwise healthy (47), suggesting that this *RAB27A* mutation may predispose to HLH *via* cooperation with other currently unknown genetic modifiers and environmental triggers. Heterozygous mutations in *RAB27A* and *STXBP2* have also been shown to mediate degranulation pathway dysfunction by acting in a dominant-negative fashion to impair CG docking and fusion (47, 48). Similarly, monoallelic mutations in *STXBP2* and *LYST* have been reported to occur with significantly greater frequency in patients with systemic juvenile idiopathic arthritis (sJIA) who develop clinical manifestations of HLH relative to those patients with sJIA who do not show signs of hyperinflammation (49). In another cohort of 175 patients with adult-onset HLH, 25 were found to have single heterozygous missense or splice-site mutations in *PRF1, MUNC13-4*, or *STXBP2*. Out of the 14 of these patients who had NK cell studies performed, nine had low or absent function. Interestingly, 12 carried the A91V allele of *PRF1*, an allele that is found at a frequency of up to 4-7% in healthy control populations (50). However, functional studies have demonstrated that expression of this allele on an otherwise WT background does confer cytotoxic dysfunction (51), and that this allele is significantly more prevalent in patients with HLH relative to healthy controls (52), suggesting that it may be a hypomorphic allele that predisposes to HLH.

Together, these data suggest that even partial genetic impairments of the degranulation pathway can be sufficient to predispose to HLH in cooperation with an adequately strong environmental stimulus and/or other genetic modifiers. Similarly, cumulative monoallelic “hits” affecting different genes within the degranulation pathway might also negatively impact immune cell function and increase the risk for HLH. In this way, patients who are doubly heterozygous for mutations in two distinct degranulation pathway genes, also known as DI, could manifest an HLH phenotype that is intermediate between one resulting from a biallelic LOF *versus* a single heterozygous LOF mutation.

At least one functional study suggests that multiple genetic hits to the degranulation pathway may contribute to a predisposition to HLH. Sepulveda et al. used murine models to study the functional consequences of combinations of heterozygous LOF in *Rab27a/Stx11, Rab27a/Prf1*, and *Rab27a/Stx11/Prf1.* In each of these mouse strains, the respective protein expression was reduced by 50%, concomitant with reduced cytotoxicity and manifestations of HLH, though the phenotypic severity differed across the combinations. Mice with the *Rab27a/Prf1* combination had a more severe phenotype than did mice with the *Rab27a/Stx11* combination, while mice with LOF of all three had the most profound NK cell dysfunction and disease severity (53), suggesting that cumulative effects on the degranulation pathway mediate hyperinflammation.

Next, we summarize available clinical data describing patients with DI of degranulation pathway genes, providing when available information regarding age of HLH onset and functional studies as correlates of the degree of degranulation pathway dysfunction.

## Case Reports and Cohort Studies

At least two case reports have described patients with HLH who were found to have monoallelic variants affecting two distinct degranulation pathway genes. One report describes a 32-year-old male who presented with fever, rash, joint pain, nausea, and vomiting and was subsequently diagnosed with adult-onset Still’s disease (54). After initial management with prednisone and antibiotics, he re-presented with fever, splenomegaly, hyperferritinemia, and hypertriglyceridemia, fulfilling four HLH diagnostic criteria. Despite further treatment with prednisone, the patient subsequently died of progressive multi-organ dysfunction. Later genetic evaluation revealed a heterozygous variant in *UNC13D* and another in *AP3B1*, though no NK cell functional studies were performed.

A second report presents the case of a 30-year-old female with chronic active Epstein Barr virus (CAEBV) infection (55). The patient fulfilled diagnostic criteria for HLH, including fever, pancytopenia, hypofibrinogenemia, hyperferritinemia, elevated soluble CD25, decreased NK cell function, and impaired CD107a mobilization, an indicator of degranulation pathway dysfunction. Treatment with etoposide and dexamethasone per the HLH-2004 protocol successfully ameliorated her hyperinflammation. Whole exome sequencing (WES) identified a heterozygous variant in *STXBP2* and another in *LYST*. Intriguingly, the patient’s mother also carried these two variants and had severely impaired NK cell function, but had never developed HLH, again suggesting that additional environmental and genetic factors may have cooperated with underlying degranulation pathway dysfunction to trigger HLH in this patient. Specifically, while the patient had a long history of CAEBV, the mother was noted to be negative for EBV, which is a well-established trigger of HLH in patients with underlying genetic predisposition (56). Through whole exome sequencing (WES), the patient was also found to carry three additional heterozygous variants affecting *LRBA* (encoding LPS-responsive beige-like anchor protein), *AIRE* (encoding autoimmune regulator protein), and *IRF8* (encoding interferon regulator factor 8). Importantly, all three of these genes have demonstrated functions in immune regulation (57–59), and the latter two variants were inherited from the patient’s father, suggesting that they may have modified the patient’s risk for developing HLH in response to an adequate immunogenic stimulus. These data underscore the importance of WES for the identification of potential genetic modifiers of underlying degranulation pathway dysfunction.

In addition to these reports, multiple groups have performed retrospective studies of cohorts of patients with HLH who underwent clinical genetic testing, most commonly *via* targeted sequencing of HLH-associated genes. Several of these studies have identified a small number of patients with heterozygous variants in two different degranulation pathway genes. In one cohort of 24 patients who were heterozygous for the A91V (c.272C>T) allele of *PRF1*, three were found to also carry heterozygous variants in *UNC13D*. Two of the three had confirmed NK cell dysfunction and presented with HLH prior to one year of age (60). Analysis of 94 Vietnamese patients identified a child presenting with HLH prior to one year of age who had single variants in both *UNC13D* and *STX11* (61). In a cohort of 14 patients with sJIA and HLH, one was found have variants in *LYST* and *STXBP2*, and had absent NK cell function and the presence of hemophagocytosis on bone marrow biopsy (49). In two cohorts of adult Chinese patients, two patients with DI of degranulation pathway variants were identified. One was found to have heterozygous variants in both *STX11* and *LYST* (62). Another had variants in *UNC13D* and *LYST*, and corresponding functional studies showed significantly impaired NK cell function and CD107a mobilization (63). Additional cohort studies presenting patients with DI of degranulation pathway genes are summarized in **Table 2**.

**Table 2:** Summary of Clinical Data of Patients with DI of Degranulation Pathway Gene Mutations.

Age at Diagnosis	Nucleotide Change	Protein Change	ClinVar Significance	PHRED Score	Reference
32 years	UNC13D c.1232G>A	p.Arg411Gln	Likely benign	22.1	(54)
	AB3B1 c.1075A>G	p.Thr359Ala	Uncertain Significance	25.6	
30 years	STXBP2 c.592A>C	p.Thr198Pro	–	24.3	(55)
	LYST c.830A>T	p.His277Leu	–	16.29	
2 months	PRF1 c.272C>T	p.Ala91Val	Conflicting IOP	24.8	(60)
	UNC13D c.I825C>T	p.Gln609X	–	5.396	
7 months	PRF1 c.272C>T	p.Ala91Val	Conflicting IOP	24.8	
	UNC13D c.2346-2349del4	p.Arg782fs	Pathogenic	–	
28 years	PRF1 c.272C>T	p.Ala91Val	Conflicting IOP	24.8	
	UNC13D c.182A>G	p.Tyr61Cys	–	–	
1 year	UNC13D c.965_967>68bp	p.Ala318X	–	–	(61)
	STX11 c.122T>C	p.Leu41Pro	–	–	
4 years	LYST c.1940T>G	p.Leu647Arg	Uncertain significance	24.2	(49)
	STXBP2 del7705108	–	–	–	
–	LYST c.7994A>G	p.Asp2665Gly	Uncertain significance	23.6	(62)
	STX11 c.842T>G	p.Phe281Cys	Uncertain significance	15.34	
18 years	LYST c.11268-5delT	–	Benign/Likely benign	–	(63)
	UNC13D c.1120C>A	p.Pro374Thr	–	–	
3 months	PRF1 c.1310C>T	p.Ala437Val	Conflicting IOP	25	(64)
	UNC13D c.169G>T	p.Glu57X	–	–	
9 months	PRF1 c.272C>T	p.Ala91Val	Conflicting IOP	24.8	
	UNC13D c.2709+6G>T	–	Benign/Likely benign	–	
11 months	PRF1 c.992C>T	p.Ser331Leu	Uncertain significance	23.8	
	UNC13D c.1232G>A	p.Arg411Gln	Likely benign	22.1	
2.25 years	PRF1 c.272C>T	p.Ala91Val	Conflicting IOP	24.8	
	UNC13D c.227C>T	p.Thr76Met	Conflicting IOP	4.913	
3 years	PRF1 c.272C>T	p.Ala91Val	Conflicting IOP	24.8	
	UNC13D c.869C>T	p.Ser290Leu	Uncertain significance	0.059	
3 years	PRF1 c.272C>T	p.Ala91Val	Conflicting IOP	24.8	
	UNC13D c.2243C>T	p.Ala748Val	Uncertain significance	18.37	
5 years	PRF1 c.1229G>A	p.Arg410Gln	Conflicting IOP	19.5	
	UNC13D c.1036G>A	p.Asp346Asn	–	–	
8 years	PRF1 c.272C>T	p.Ala91Val	Conflicting IOP	24.8	
	UNC13D c.3160A>G	p.Ile1054Val	Uncertain significance	10.51	
9 years	PRF1 c.10C>T	p.Arg4Cys	Conflicting IOP	0.163	
	UNC13D c.3232G>C	p.Ala1078Pro	–	23.2	
9 years	PRF1 c.272C>T	p.Ala91Val	Conflicting IOP	24.8	
	UNC13D c.2896C>T	p.Arg966Trp	Benign/Likely benign	26.3	
10 years	PRF1 c.272C>T	p.Ala91Val	Conflicting IOP	24.8	
	UNC13D c.2896C>T	p.Arg966Trp	Benign/Likely benign	26.3	
12 years	PRF1 c.50delT	p.Leu17fs	Pathogenic	–	
	UNC13D c.1579C>T	p.Arg527Trp	Benign/Likely benign	21.3	
13 years	PRF1 c.445G>A	p.Gly149Ser	Pathogenic	23.7	
	UNC13D c.2896C>T	p.Arg966Trp	Benign/Likely benign	26.3	
13 years	PRF1 c.272C>T	p.Ala91Val	Conflicting IOP	24.8	
	UNC13D c.2896C>T	p.Arg966Trp	Benign/Likely benign	26.3	
5 years	PRF1 c.272C>T	p.Ala91Val	Conflicting IOP	24.8	
	STXBP2 c.1034C>T	p.Thr345Met	Benign/Likely benign	25.6	
10 years	PRF1 c.272C>T	p.Ala91Val	Conflicting IOP	24.8	
	STXBP2 c.1034C>T	p.Thr345Met	Benign/Likely benign	25.6	
16 years	PRF1 c.655T>A	p.Tyr219Asn	–	28.6	
	STXBP2 c.1034C>T	p.Thr345Met	Benign/Likely benign	25.6	
21 years	PRF1 c.272C>T	p.Ala91Val	Conflicting IOP	24.8	
	STXBP2 c.1586G>C	p.Arg529Pro	Conflicting IOP	26	
24 years	PRF1 c.50delT	p.Leu17fs	Pathogenic	–	
	STXBP2 1459G>A	p.Val487Met	Likely benign	23	
2 months	UNC13D c.2896C>T	p.Arg966Trp	Benign/Likely benign	26.3	
	STXBP2 c.911C>T	p.Thr304Met	Uncertain Significance	26.2	
5 months	UNC13D c.1389+1G>A	–	Pathogenic	–	
	STXBP2 c.1782*12G>A	–	–	–	
8 months	UNC13D c.2828A>G	p.Asn943Ser	Likely benign	25.7	
	STXBP2 1782*12G>A	–	–	–	
1 year	UNC13D c.2828A>G	p.Asn943Ser	Likely benign	25.7	
	STXBP2 c.715C>T	p.Pro239Ser	–	23.9	
2 months	UNC13D c.2030T>C	p.Ile677Thr	–	26.4	
	STX11 c.221C>T	p.Thr74Met	Conflicting IOP	26.2	
14 years	STXBP2 c.568C>T	p.Arg190Cys	Conflicting IOP	31	
	STX11 c.9C>A	p.Asp3Glu	Uncertain significance	23.8	
5 years	STXBP2 c.1034C>T	p.Thr345Met	Benign/Likely benign	25.6	
	RAB27A c.295T>G	p.Phe99Val	–	28.7	
1 year	LYST c.11268-5delT	–	Benign/Likely benign	–	(65)
	STXBP2 c.1474G>A	p.Asp492Asn	–	29.7	
2 years	LYST c.4732G>A	p.Ala1578Thr	–	–	
	UNC13D c.2341G>A	p.Val781Ile	Conflicting IOP	0.764	
7 years	LYST c.11268-5delT	–	Benign/Likely benign	–	
	UNC13D c.2917A>G	p.Lys973Glu	Uncertain significance	23.1	
15 years	LYST c.10688C>T	p.Ser3563Leu	–	32	
	PRF1 c.655T>A	p.Tyr219Asn	–	28.6	
16 years	LYST c.11268-5delT	–	Benign/Likely benign	–	
	UNC13D c.2896C>T	p.Arg966Trp	Benign/Likely benign	26.3	
17 years	LYST c.10800+4G>T	–	Conflicting IOP	–	
	STX11 c.9C>A	p.Asp3Glu	Uncertain significance	23.8	
–	LYST c.4265C>T	p.Ala1422Val	Uncertain significance	22.2	
	RAB27A c.418C>G	p.Gln140Glu	Conflicting IOP	18.45	
1 year	PRF1 1349C>T	p.Thr450Met	Pathogenic	23.5	(66)
	AP3B1 c.1321A>G	p.Ile441Val	–	–	
25 years	PRF1 c.272C>T	p.Ala91Val	Conflicting IOP	24.8	(50)
	STXBP2 c.795-4C>T	–	Benign/Likely benign	–	

Summary of age at HLH onset and identified degranulation pathway variants for 44 patients reported in published case reports and cohort studies. “-” indicates information not available, IOP – interpretations of pathogenicity.

In the largest study to date directly evaluating the plausibility of DI in HLH, Zhang et al. analyzed targeted sequencing data from 2,701 patients with HLH and identified 28 with heterozygous variants in two different degranulation pathway genes. Twenty-one of these patients had variants in *PRF1* plus another gene, while the remaining seven had variants in two other genes in the degranulation pathway. These seven tended to have an earlier age of onset, comparable to that of patients with classic biallelic LOF mutations. Of the four who had corresponding functional studies, three had impaired CD107a mobilization. Based on these data, the authors conclude that cooperation between two distinct variants likely mediates immune dysregulation that is sufficient to cause HLH, though they acknowledge the caveat of incomplete functional data (64).

Finally, in a cohort of 48 pediatric patients with HLH who underwent WES, Chinn et al. identified heterozygous variants in two degranulation pathway genes in seven (14.5%) patients. To determine the significance of these findings, the authors compared the genotypes observed in their patient cohort to genotypes present in the Baylor-Hopkins CMG database. Intriguingly, they concluded that these combinations were unlikely to occur more commonly in patients with HLH than in the general population, and based on this result, cautioned that other studies reporting on the clinical significance of DI should be interpreted carefully (65).

The data from these case reports and cohort studies are summarized in **Table 2**. To enable comparisons between these studies, we used ClinVar (67) and CADD scores (68), reported as PHRED-scaled scores, to determine the significance of the reported variants as known at the time of publication of this article.

## Concluding Remarks and Future Directions

Here, we review the consequences of LOF mutations in genes encoding several key components of the lymphocyte degranulation pathway, providing a mechanistic basis for interpreting the clinical significance of variants in one or more of these genes. Additionally, we summarize the clinical and experimental data that support revisiting the historical division of HLH into its familial and secondary forms. These data instead favor a more comprehensive model that takes into account both genetic and environmental factors, resulting in a threshold over which the combined effect of these two factors can lead to the development of HLH in the context of an appropriately strong antigenic stimulus. In support of this model, we present an analysis of 44 patients with HLH lacking the classic biallelic LOF mutations but found instead to harbor heterozygous variants in two different genes within the degranulation pathway (**Table 2**).

While the clinical data presented here suggest the plausibility of DI in HLH, several caveats limit the interpretation of these data. First, of the variants identified in these studies that have information available in the ClinVar database, 33% are reported as “benign” or “likely benign” and 58% have “uncertain significance” or “conflicting interpretations of pathogenicity”. Without corresponding functional data, it is not possible to determine their true impact on protein expression or activity. In addition, clinical NK cell function and CD107a mobilization studies are lacking for many of these patients, further complicating the interpretation of any putative functional impacts of the reported genotypes. Second, most of the studies summarized here relied on targeted sequencing of HLH-associated genes rather than on WES, precluding the ability to identify other potential genetic causes of immune dysregulation. Finally, as noted by Chinn et al. (65), many of the reported digenic combinations are also present at some frequency in healthy populations, confounding the attribution of these genotypes to the development of HLH in these patients. Taken together, the data presented here suggest that co-inheritance of variants in degranulation pathway genes does occur in a subset of patients who develop HLH. However, more comprehensive studies consisting of WES in combination with functional and biochemical analyses of the associated variants and correlation with environmental triggers will be required to better understand the impact of these variant combinations on predisposition to HLH and the extent to which these combinations manifest clinically as pseudo-DI or true DI.

## Author Contributions

ES and LM wrote the manuscript. MH and KN reviewed and edited the manuscript. All authors approved the submitted version.

## Funding

This work was supported by grants from the Immune Deficiency Foundation (MH) and the University of California, San Francisco Sandler Program for Breakthrough Biomedical Research (MH). LM is supported by the NIGMS Medical Scientist Training Program Grant T32GM141323.

## Conflict of Interest

MH is a consultant for Novartis and Sobi. KN receives research funding from Incyte.

The remaining authors declare that the research was conducted in the absence of any commercial or financial relationships that could be construed as a potential conflict of interest.

## Publisher’s Note

All claims expressed in this article are solely those of the authors and do not necessarily represent those of their affiliated organizations, or those of the publisher, the editors and the reviewers. Any product that may be evaluated in this article, or claim that may be made by its manufacturer, is not guaranteed or endorsed by the publisher.
